# Computer-assisted preoperative planning of bone fracture fixation surgery: A state-of-the-art review

**DOI:** 10.3389/fbioe.2022.1037048

**Published:** 2022-10-14

**Authors:** Jet Zoë Moolenaar, Nazli Tümer, Sara Checa

**Affiliations:** ^1^ Berlin Institute of Health at Charité, Universitätsmedizin Berlin, Julius Wolff Institute, Berlin, Germany; ^2^ Department of Biomechanical Engineering, Delft University of Technology (TU Delft), Delft, Netherlands

**Keywords:** bone fracture fixation, osteosynthesis, preoperative planning, computer-assisted, virtual surgery

## Abstract

**Background:** Bone fracture fixation surgery is one of the most commonly performed surgical procedures in the orthopedic field. However, fracture healing complications occur frequently, and the choice of the most optimal surgical approach often remains challenging. In the last years, computational tools have been developed with the aim to assist preoperative planning procedures of bone fracture fixation surgery.

**Objectives:** The aims of this review are 1) to provide a comprehensive overview of the state-of-the-art in computer-assisted preoperative planning of bone fracture fixation surgery, 2) to assess the clinical feasibility of the existing virtual planning approaches, and 3) to assess their clinical efficacy in terms of clinical outcomes as compared to conventional planning methods.

**Methods:** A literature search was performed in the MEDLINE-PubMed, Ovid-EMBASE, Ovid-EMCARE, Web of Science, and Cochrane libraries to identify articles reporting on the clinical use of computer-assisted preoperative planning of bone fracture fixation.

**Results:** 79 articles were included to provide an overview of the state-of-the art in virtual planning. While patient-specific geometrical model construction, virtual bone fracture reduction, and virtual fixation planning are routinely applied in virtual planning, biomechanical analysis is rarely included in the planning framework. 21 of the included studies were used to assess the feasibility and efficacy of computer-assisted planning methods. The reported total mean planning duration ranged from 22 to 258 min in different studies. Computer-assisted planning resulted in reduced operation time (Standardized Mean Difference (SMD): -2.19; 95% Confidence Interval (CI): -2.87, -1.50), less blood loss (SMD: -1.99; 95% CI: -2.75, -1.24), decreased frequency of fluoroscopy (SMD: -2.18; 95% CI: -2.74, -1.61), shortened fracture healing times (SMD: -0.51; 95% CI: -0.97, -0.05) and less postoperative complications (Risk Ratio (RR): 0.64, 95% CI: 0.46, 0.90). No significant differences were found in hospitalization duration. Some studies reported improvements in reduction quality and functional outcomes but these results were not pooled for meta-analysis, since the reported outcome measures were too heterogeneous.

**Conclusion:** Current computer-assisted planning approaches are feasible to be used in clinical practice and have been shown to improve clinical outcomes. Including biomechanical analysis into the framework has the potential to further improve clinical outcome.

## 1 Introduction

### 1.1 Background

Bone fractures are the most common form of hospitalized trauma and result in a significant healthcare burden. A systematic analysis from the Global Burden of Diseases, Injuries, and Risk Factors Study (GBD) 2019 has estimated the global number of new fractures to be 178 million (Wu et al., 2021). Since fracture rates are increasing with age, the GBD 2019 predict that this number will increase even further in the coming years considering the ageing of the population. Fractures are especially common among people with osteoporosis and are associated with an annual cost of 37.5 billion euros and a loss of one million quality-adjusted life years in Europe (Borgström et al., 2020). Although bone has the ability to self-generate, fracture healing complications occur frequently and non-union rates of 5–10% of all fractures have been reported (Ekegren et al., 2018).

Surgical treatment of bone fractures aims to restore the original anatomy of the bone in order to recover the lost motor function and is one of the most frequent surgical procedures in the orthopaedic field (Yoshii et al., 2021). The surgical treatment typically consists of two steps. First, the fragments are surgically reduced to their original anatomical sites and second, the bone is stabilized using fixation tools such as screws, nails, and plates. The fixation of the bone, also called osteosynthesis, is done to create the appropriate mechanical and biological conditions for bone healing. Suboptimal reduction and fixation can cause delayed bone union, traumatic arthritis (Olson et al., 2012), re-dislocation of fractures, and mal-/non-unions (Yoshii et al., 2021). Revision surgery is required in 10–15% of all cases (Kovler et al., 2015). In intra-articular fractures, exact reduction of the joint congruency is an important factor to avoid the development of post-traumatic arthrosis (Citak et al., 2011; Maini et al., 2018; Casari et al., 2021).

There are typically many options for fracture fixation and the optimal fixation is highly dependent on the bone geometry and fracture pattern. Therefore, the surgical treatment of bone fractures calls for detailed preoperative planning. Conventionally, the planning of bone fracture fixation surgery is done using plain radiographs, tracing paper, and view boxes (Mast et al., 1989; Messmer et al., 2001a; Pilson et al., 2008). The surgeons then typically decide on the optimal treatment based on experience and habitual practices of the department (Madeja et al., 2021; Yoshii et al., 2021). Benefits are that this conventional approach is simple, familiar to surgeons, fast, low cost, and leads to a low radiation exposure for the patients (Chen et al., 2020; Yoshii et al., 2021). However, based on 2D radiographs, it is difficult to determine rotational reduction and inaccuracies may arise due to differences in scales of radiographic images (Yoshii et al., 2019b). Additionally, this conventional method often fails to provide surgeons with a clear understanding of the fracture pattern, especially in complex fracture situations (He et al., 2022). This means that the surgical plan might need to be adjusted intra-operatively and implants often need to be contoured during surgery, possibly leading to longer operation times, intra-operative bleeding, and intra-operative fluoroscopic exposure times.

Computer-assisted preoperative planning (CAPP), or virtual surgical planning (VSP), may resolve many of these issues associated with conventional planning. In the field of fracture fixation surgery, VSP usually includes: 1) obtaining a three-dimensional (3D) anatomic reconstruction of the fractured bone, 2) virtual bone fragment reduction, and 3) virtual fixation of the fragments using implants (Jiménez-Delgado et al., 2016). Possible benefits of VSP include:

1) Increased understanding of the fracture characteristics such as the direction of the fracture lines, size of the fracture, and the number and location of fragments (Boudissa et al., 2018).
2) Avoid extensive dissection and soft tissue stripping, repeated manipulations of reduction fixation, possibly leading to shorter operation time and less bleeding (Hung et al., 2019; Zheng et al., 2022).
3) Choose optimal operative approach, e.g*.*, appropriate choice of screw lengths and plate sizes (Yoshii et al., 2019b).
4) Automatic contouring or pre-bending of fixation devices and design of drilling guides (Merema et al., 2017).
5) Practice surgery virtually and decrease learning curve for surgeons (Wang D. et al., 2020).
6) Better doctor-patient communication about preoperative plan (Wang et al., 2017).

VSP is increasingly available to orthopedic surgeons (Yoshii et al., 2019a) and is routinely deployed in orthopedic surgery mostly for arthroplasties, deformity correction surgery, spinal fusion surgery, and fracture revision surgeries (Vetter et al., 2014; Székely et al., 2016; Takao et al., 2018a). Until recently, VSP has lagged behind in trauma cases, which are more time-critical (Nilsson et al., 2021).

### 1.2 Rationale

Despite existing reservations, many studies suggest that VSP of bone fracture fixation is feasible to be employed in clinical practice and it improves clinical outcomes. However, the clinical efficacy and feasibility of existing VSP systems for bone fracture fixation have never been systematically reviewed. Previous reviews have focused on the description and technical details of the involved steps (Jiménez-Delgado et al., 2016) or on a specific type of fracture treatment, such as acetabular fracture surgery (Boudissa et al., 2018). Banierink et al. (2021) and Assink et al. (2021) have both reviewed whether ‘3D-assisted surgery’ improves clinical outcomes when used for the planning of pelvic ring fractures and tibial plateau fractures, respectively. However, these reviews focus on different concepts of 3D-assisted surgery, including 3D virtual visualization, 3D printed hand-held fracture models, pre-contouring of osteosynthesis plates, 3D printed surgical guides, and intra-operative 3D imaging, rather than computer models to assist in planning. Additionally, they focus on one fracture type only and do not assess existing systems in terms of their clinical applicability and potential improvements.

### 1.3 Objectives

The aim of this review is to assess the clinical applicability of computer-assisted preoperative planning of bone fracture fixation surgery. This will be done by 1) providing a comprehensive overview of the state-of-the-art approaches including existing software systems and design gaps, 2) assessing the clinical feasibility of the existing approaches, and 3) assessing the clinical efficacy of the existing approaches in terms of clinical outcomes as compared to conventional planning methods.

The clinical feasibility will be assessed in terms of the required input data, segmentation methods and duration, virtual fracture reduction methods and duration, and virtual fracture fixation methods and duration. The assessed clinical outcomes will include differences in operation time, blood loss, fluoroscopy frequency, reduction quality, functional outcomes, postoperative complications, and fracture healing time between computer-assisted and conventional planning.

This paper is organized as follows. First, the literature search strategy and results are presented in Section 2 and Section 3. Next, the overall process of computer-assisted preoperative planning of bone fracture fixation is described in Section 4. Here, the different stages, including different approaches for each stage, limitations, and new trends are described. In Section 5, existing clinically applied software solutions will be presented, and in Section 6 and Section 7, the clinical feasibility and efficacy of the VSP approaches are investigated as compared to conventional planning methods. Finally, in Section 8, the results are discussed and conclusions are presented in Section 9.

## 2 Literature search

The literature search was performed according to the Preferred Reporting Items for Systematic Reviews (PRISMA) (Page et al., 2021). This means that a systematic search strategy was developed and used in multiple databases. Thereafter, eligible studies were selected based on inclusion and exclusion criteria that were formulated prior to study selection. Articles were assessed for eligibility by one reviewer.

### 2.1 Search strategy and databases

**FIGURE 1:**
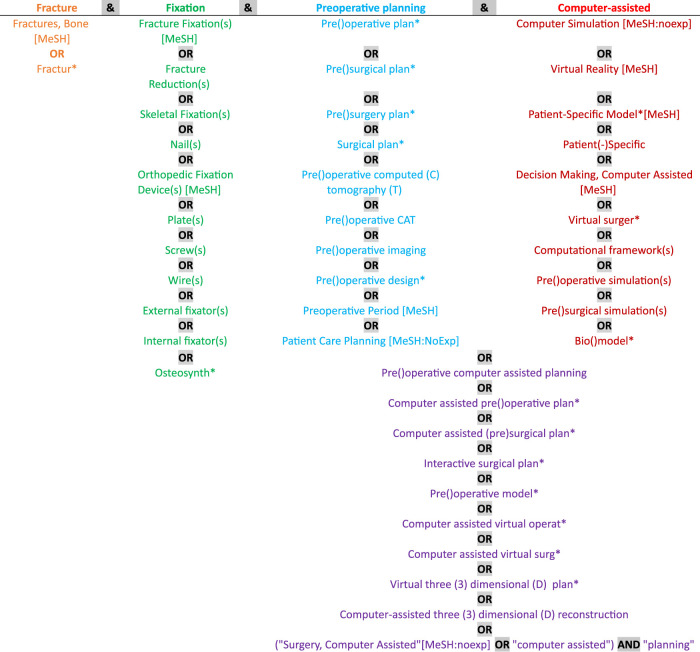
Schematic view of the query construction. Columns: aspects. Rows: synonyms.

The MEDLINE-PubMed, Ovid-EMBASE, Ovid-EMCARE, Web of Science, and Cochrane libraries were searched on March 3rd of 2022 without a limit on the publication date. The search strategy was developed in collaboration with an experienced medical librarian. It was developed to identify studies related to computer-assisted preoperative planning of bone fracture fixation. These aspects, including different ways to describe the aspect, were combined to construct the used search strings. Figure 1 shows a schematic view of the query construction. The columns represent the four aspects of the query. Each row contains different ways (i.e., synonyms) to describe the aspect. The terms are used as text words and–in the case of PubMed–as Medical Subject Headings (MeSH) terms too when indicated. The string was developed while making sure that certain key references were found using the search string. For each database, the string was slightly adapted to be suitable for the controlled vocabularies of the database.

### 2.2 Study selection: Eligibility criteria

To provide a comprehensive overview of the state-of-the-art in computer-assisted preoperative planning of bone fracture fixation, studies were only included if they reported on clinically applied virtual planning of bone fracture fixation. This means that studies were excluded if they:

1) were not clinically applied^1^
  1) Animal studies
  2) Cadaver studies
  3) Retrospective simulation studies
2) did not focus on bone fracture fixation
  1) Deformity corrections
  2) Arthroplasties
  3) Spinal fusion
  4) Orbital floor reconstructions
3) did not use a virtual model (i.e., use of a 3D printed model without any computer-assistance)
4) did not focus on preoperative planning
  1) Only intra-operative navigation
  2) Studies about the classification of injuries by means of computer models

Additionally, studies that were not available in English were excluded.

To analyze the efficacy and feasibility of existing software approaches, additional inclusion criteria were formulated. Studies were included for this analysis if they had a control group and reported on 1) simulation times and/or 2) clinical outcomes.

### 2.3 Analysis and data extraction

General study characteristics, investigated systems, fracture classification and intervention, input data, segmentation methods and duration, virtual reduction methods and duration, virtual fixation methods and duration, analysis methods and duration, clinical outcome measures, and main conclusions were extracted for the included studies with a control group. Studies without a control group were only screened for the investigated system and fracture type to get an overview of the existing systems.

### 2.4 Statistical analysis

Analysis of the extracted clinical outcomes (i.e., operation time, blood loss, fluoroscopy frequency, fracture healing time, hospitalization duration and postoperative complications) was performed in Review Manager (version 5.4.1). Continuous variables such as operation times, the amount of blood loss, fluoroscopy frequency, fracture healing time and duration of hospitalization are presented as means plus standard deviations. Standardized mean differences (SMD/Cohen’s d) were computed, since the scales of the reported outcomes are considerably different. A random effects model was used since the studies have been conducted under dissimilar conditions and for different fracture types. The postoperative complication rates are presented as frequencies and risk ratios (RR) were computed, i.e., the ratio of the complication probability in the VSP group to the probability in the conventional group.

## 3 Literature search results

### 3.1 Study selection

**FIGURE 2:**
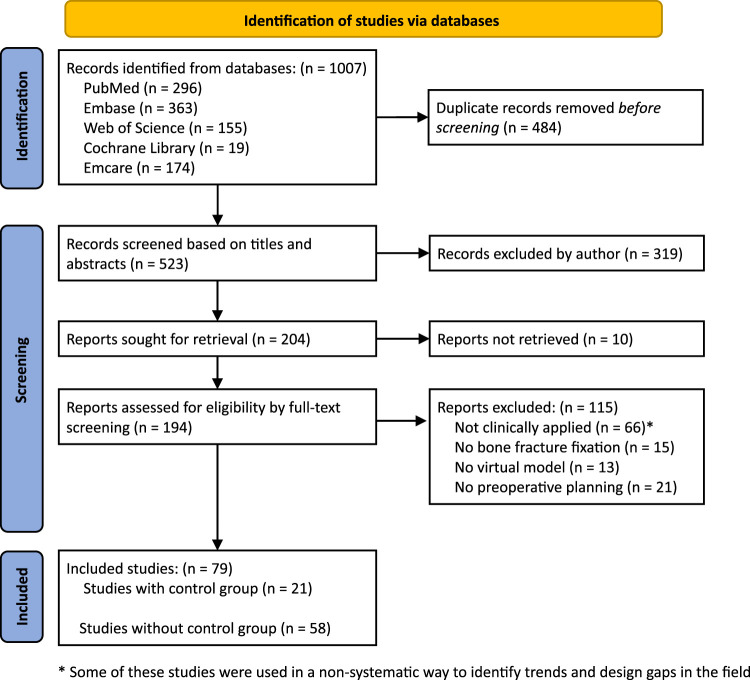
PRISMA flow diagram of literature search results.

The search yielded a total of 1,007 studies, and after the removal of duplicates, 523 studies were screened based on title and abstract for eligibility. Based on this, 319 studies were excluded, and the remaining 204 records were sought for retrieval. 10 records were not retrieved or were not available in English. Consequently, 194 reports were assessed for eligibility based on full-text screening. 115 records were excluded based on the exclusion criteria outlined in Section 2.2. Out of these 115 records, 66 articles did discuss computer-assisted preoperative planning of bone fracture fixation, but the discussed systems were not clinically applied, either because the systems are still experimental or because they concerned a review article. However, some of these articles were useful to gain an overview of the trends in the field, and some of them will be used to present an overview of the state-of-the-art in the coming sections (objective 1). However, only the remaining 79 were systematically analyzed. Out of these 79 studies, 21 studies had a control group and were used to assess the efficacy and feasibility of the existing approaches for computer-assisted preoperative planning of bone fracture fixation (objectives 2 and 3). The remaining 58 studies were clinically applied without a control group and were only screened for the investigated system and fracture type to give an overview of existing approaches (objective 1). The study selection is shown schematically in the PRISMA flow diagram in Figure 2.

## 4 Stages of computer-assisted preoperative planning

**FIGURE 3:**
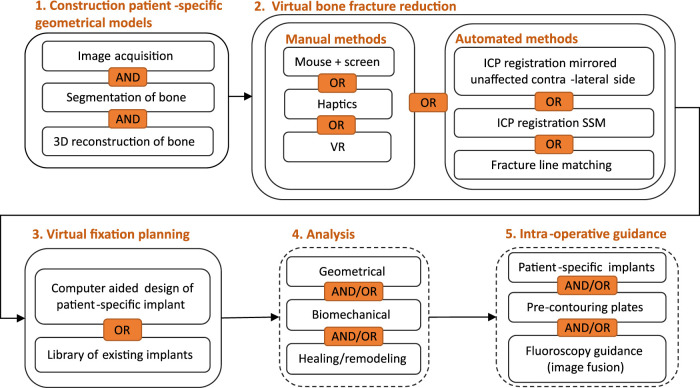
Flow diagram of different stages of computer-assisted preoperative planning of bone fracture fixation, including different approaches for each stage. VR, virtual reality; ICP, iterative closest point; SSM, statistical shape model.

Computer-assisted preoperative planning of bone fracture fixation typically consists of the following stages: 1) generation of patient-specific geometrical models, 2) virtual bone fracture reduction, 3) virtual bone fracture fixation, and 4) analysis of surgical planning. To make sure the surgery is done according to planning, 5) intra-operative guidance may be provided. Alternatively, 3D printed models are sometimes used as well for some stages of the planning. Each of these stages is detailed in the following sections. An overview of the stages is given in Figure 3. Jiménez-Delgado et al., 2016 discuss these stages in more technical detail.

### 4.1 Construction of patient-specific geometrical models

The first step in VSP is the construction of a 3D patient-specific model that represents the bone and bone fragments involved in the fracture. In general, these geometrical models can be made using two main approaches:

The first approach–which is, according to the authors’ knowledge, the only one currently used in clinical practice—requires the application of 3D medical imaging technologies, such as computed tomography (CT). The second approach is to use statistical shape models (SSMs), which provide a way to generate an average bone model including the main modes of variation of a given population (Sarkalkan et al., 2014). These two approaches are elaborated on in the following subsections.

#### 4.1.1 Approach 1: Using 3D medical imaging technologies

After image acquisition using 3D medical imaging technologies (Figure 3, step 1.1), the bone should be segmented (Figure 3, step 1.2) from each slice. This is a relatively complex task since bone consists of two types of tissues with different properties and intensities on the CT scan: cortical tissue, which is very dense, and trabecular tissue, which is more heterogeneous and typically has a lower intensity on CT (Jiménez-Delgado et al., 2016). Additionally, the intensity of the bone might vary between slices and patients. This task is therefore hard to automate and often requires manual user-interaction for refinement. There are different techniques to segment bone from CTs, including thresholding, region growing, watershed and registration methods using atlases (Jiménez-Delgado et al., 2016). After segmentation, the segmented slices are stacked to create a 3D reconstruction (Figure 3 step 1.3). For the subsequent steps, it is often necessary to generate a mesh (i.e., a collection of vertices, edges, and faces) from such a CT stack, e.g., for automated reduction methods (Figure 3, step 2) and analysis (Figure 3, step 4). For mesh generation, the marching cubes algorithm, as introduced by Lorensen and Cline (1987) is most widely used (Fornaro et al., 2010a; Boudissa et al., 2021b; Casari et al., 2021; Zindel et al., 2021). The outlined methods are often integrated in commercial medical imaging software such as Materialise Mimics (Materialise, Leuven, Belgium) AMIRA (ThermoFisher, Berlin, Germany) or SimpleWare (Synopsys, CA, USA). Moreover, some open-source software such as 3D Slicer^2^ (Fedorov et al., 2012) and ImageJ/Fiji^3^ (Schneider et al., 2012) offer similar capacities.

After 3D reconstruction, the bone model typically needs post-processing to separate the fracture fragments. This fragmentation is especially challenging in comminuted fractures where fragments might overlap. Typically, the separation is already integrated in the segmentation procedure, by manually editing the used segmentation masks in the 2D slices (Hu et al., 2011; Maini et al., 2018; Boudissa et al., 2021b; Zheng et al., 2022). In other (experimental) studies, the separation of fragments is done semi-automatically in 3D. For example, Wang G. Y. et al. (2016) used an approach where the surgeon approximately marked the boundary of two fragments on the 3D model and thereafter, the software automatically defined the fragment borders. A different approach was developed by Buschbaum et al. (2015), who used an algorithm to detect strongly curved edges to generate fracture lines and separate the fragments. This approach was evaluated using artificial plastic SYNBONE models with fractures and was shown to work on cylindrical fragments but performed poorly at fractures near joints. Yoshii et al. (2020) also separated the fragments automatically according to the fracture lines and showed that imaging slices of less than 2 mm are required to reach enough accuracy. A multiple region growing method with an automatic seed-point assignment was used by Lee et al. (2014) to separate the fragments. This approach was evaluated by means of a retrospective simulation study and was shown to perform well, but occasionally required manual intervention by an expert. Liu et al. (2019) used the snake (active contour) method to automatically separate the broken bones. Voss et al. (2016) used a semi-automatic landmark-based approach to separate fragments. In this approach, the user has to indicate landmarks in regions where segments remained connected and landmark pairs describing the gap. A 3D circular cutting template, adapted according to these landmarks, was then computed to separate the fragments. After fragment separation, the model is usually further post-processed using smoothing and/or remeshing techniques (Thomas et al., 2011; Han et al., 2019; Boudissa et al., 2021b). This can be done using commercial software such as Materialise Mimics (Zhang et al., 2015), but also using non-commercial software such as MeshLab (Boudissa et al., 2021b).

Currently, this stage of the planning requires a high amount of time, manual user interaction and sometimes, expert knowledge. All of the commercially available VSP software rely on this approach using segmentation of medical CT images (Vitković et al., 2018). In Section 6, the used image acquisition parameters, segmentation methods and segmentation duration are documented for the included controlled studies. Advancements in machine learning methods for automated segmentation might reduce the required time for this process (He et al., 2022). Another intrinsic drawback of using this image-based approach is the radiation exposure it causes for the patient. Because of this, CT scans of the whole bone are usually not done for simple fractures in current clinical practice. Often, CT imaging data is only available of the fracture region.

#### 4.1.2 Approach 2: Using statistical shape models

An alternative approach for the creation of 3D geometrical models is to use SSMs or atlases of bones. A personalized model of the given patient can then be created using either 1) parametric functions to correlate patient-specific characteristics to certain modes of variations or 2) using two-dimensional (2D) imaging modalities.

These patient-specific characteristics can be either simple morphometric parameters that can be read from X-ray radiographs (the Method of Anatomical Features) (Majstorovic et al., 2013; Vitković et al., 2018) or other patient-specific characteristics such as gender, age, and BMI (Klein et al., 2015). Klein et al. (2015) developed a parametric SSM of the femur as a function of age, BMI, and femur length as part of an effort to develop finite element (FE) models with geometries that are parametric with subject characteristics. The average error in the cortical bone area between the predicted geometries and a validation set of cadaver femur geometries across five shaft locations was 2.9% (Klein et al., 2015). However, in this study, fractures were not considered. One of the current limitations of this approach is that the SSM should be fit to the patient-specific fracture pattern although the model does not capture this information.

It has also been proposed to use 2D imaging modalities and 2D to 3D atlas-based registration methods to reconstruct a 3D model based on two orthogonal 2D radiographic images. Messmer et al. (2001a, 2001b) proposed to use two orthogonal 2D images of the contralateral limb to find the most similar 3D model of the intact bone. The surgeon was then able to draw the fracture pattern manually on the 3D model and create the virtual fracture interactively based on the X-ray of the fractured bone. Schumann et al., 2016 developed a methodology that requires two orthogonal X-ray images and a 3D SSM of the bone to be reconstructed. The SSM was iteratively fit to X-ray based features. A final non-rigid deformation was applied to reconstruct the bone geometry. They also included a methodology to reconstruct two bone fragments by splitting up the SSM into two fragments using an estimate of the fragment lengths. However, these approaches are only suitable for simple fractures.

### 4.2 Virtual bone fracture reduction planning

After the construction of a 3D geometrical model of the fractured bone, virtual bone fracture reduction is typically performed. This is the process by which the bone fragments are rotated and translated to recover their original anatomical position. There are different methods to achieve this virtual reduction. The most common method is by free-hand reduction of the bone fragments using manual translation and rotation through computer mouse interaction (Fornaro et al., 2010b; Maini et al., 2018; Chen et al., 2020; Boudissa et al., 2021b). The commercially available VSP systems used in clinical practice also employ this methodology (see Section 6).

It is also possible to manually reduce the bone fragments using more advanced interfaces such as haptic systems or virtual reality (VR) environments (Fornaro et al., 2007; Fornaro et al., 2010a; Schvartzman et al., 2014; Kovler et al., 2015; Girod et al., 2016; Choi and Schmutz, 2020; Nilsson et al., 2021). This has been shown to be especially helpful for training purposes of surgical procedures, since the surgeon performing the reduction gets realistic feedback (Schvartzman et al., 2014; Choi and Schmutz, 2020). Schvartzman et al. (2014) and Girod et al. (2016) developed a VSP system for craniofacial fractures featuring bimanual touch and force feedback. The system was evaluated by three surgeons, and they reported that the tool was easy and comfortable to use in daily clinical practice. Compared to mouse-based interfaces, the haptic system was rated higher in terms of intuitiveness and self-reported quality of repair and haptic simulation results were closer to actual postoperative outcomes. The user interaction time was approximately 15 min.

Many automated reduction methods have also been proposed and tested in experimental settings only. The first class of methods relies on the incorporation of the mirrored healthy contralateral bone to facilitate reduction. The in-house developed software CASPA (Balgrist, CARD AG, Switzerland) was used by Casari et al. (2021) to plan intra-articular radius fracture fixation and used iterative closest point (ICP) registration of the fractured model to the mirrored healthy contralateral radius. Zhao et al. (2021) used the same ICP approach for fractures of the pelvis and found that the method achieved an average global distance error below 4 mm when applied to artificially fractured models, which is excellent according to the criteria for reduction precision of pelvic fractures introduced by Matta and Tornetta (1996). Okada et al. (2009) applied the same method to broken femur heads but found that this did not lead to satisfactory results due to poor initialization. Furnstahl et al. (2012) proposed a registration algorithm to the mirrored contralateral healthy bone compromised of local and global registration steps for the proximal humerus to overcome this problem.

This mirroring method relies on the assumption that the contralateral bones are symmetric, which is often not true in case of natural shape differences or bilateral trauma (Han et al., 2021). Therefore, another approach has been proposed which is based on the use of a SSM to serve as a template for registration of the fracture fragments. Han et al. (2021) used an adaptive SSM template to describe shapes and poses of pelvic bones and used a registration scheme to simultaneously solve for reduction planning transformations and SSM shape and pose parameters. The approach was shown to accurately perform virtual pelvic fracture reduction in a number of simulation cases and performed significantly better than the mirror method. Ead et al. (2021) also used SSMs as templates when constructing unilaterally fractured pelvises. The fracture fragments were registered to the template using the coherent point drift method. The average root-mean-square-error between reconstructed and intact hemipelves was less than 2 mm.

The last automated virtual reduction method is based on the matching of the fracture lines of the different fragments. Many authors have attempted to apply this method but often struggle with challenging fracture line definitions. Kovler et al. (2015) used a semi-automatic approach where the surgeon had to interactively mark the bone fragment surfaces bringing the bone fragments into coarse alignment. An ICP rigid registration algorithm was then applied to the fracture lines to perform fine alignment. They tested the approach using a virtual fracture on a healthy bone model and found a final mean target registration error of 1.79 mm with a run time of 3 min. However, true fractures might suffer from more complex fracture lines, which are harder to match. Buschbaum et al. (2015); Buschbaum et al. (2017) also computed the target position for the alignment based on the fracture patterns. Fracture lines were extracted by thresholding for strongly convex curved edges. The ICP algorithm was then used to calculate the required transformation aligning the fragments. In their later work (Buschbaum et al., 2017), they used this transformation as target position and then computed the optimal collision-free reduction pathway of minimal force using a path search algorithm (modified A*-algorithm (Hart et al., 1968)) considering musculoskeletal forces. When applied on five broken femoral SYNBONE models, they found a highest translational error of less than 2 mm and successful path planning was achieved in every case in a period of 84.62 ± 74.11 s. Thomas et al. (2011) used ICP to match the fracture lines as well and found that an initial alignment using registration to an intact template (i.e., the contralateral unaffected bone) greatly improved alignment speed and stability. Simulations for ten tibial plafond fracture cases achieved an average alignment error of 0.39 ± 0.5 mm. Liu et al. (2019) first roughly aligned the bone fragments using automatic long bone axis detection by principal component analysis (PCA), then aligned the principal directions of the cross-section vertices and then did fine registration on the cross-section vertex set using ICP. Using eight broken goat tibias, the framework was shown to have an error of maximally 2.9 mm within a period of 107 s (including automatic segmentation, 3D bone reconstruction and fracture reduction). Paulano-Godino and Jiménez-Delgado (2017) developed an automatic method to calculate the contact zone between bone fragments using a discretized sweep on the bone point cloud and filtering of the candidate points based on the distance from each point to the opposite fragment and the estimated curvature at each point. Matching of the contact points was then performed using ICP. When applied to the tibia, they found a translation error of 1.61 mm compared to manual alignment by an expert within a period of 74.87 s.

It is also possible to directly use the mirrored contralateral healthy bone or an SSM template for the design of patient-specific implants, leaving out the virtual fracture reduction step. This has been done by *inter alia* Agarwal et al. (2021).

### 4.3 Virtual bone fracture fixation planning

After virtual fracture reduction, the next phase is typically to virtually place a fixation device to stabilize the fracture. There are different approaches to do this. The implants can either be chosen from a library of existing implants (Joskowicz et al., 1998; Chen et al., 2014; Schvartzman et al., 2014; Huang et al., 2015; Xia et al., 2015, 2019) and scaled if necessary, or implants can be designed for each case independently using computer-aided design (CAD) tools (Wang et al., 2017; Badiali et al., 2020; Ramanathan et al., 2020). In case of patient-specific CAD implants, they are often 3D printed afterwards to be used in surgery. In some studies, implants are not actually virtually placed, but important geometric parameters - such as plate sizes and screws lengths and angles–are decided upon based on the virtually reduced model using measuring tools. Another option is to pre-contour the implants based on a 3D printed model of the virtually reduced model (see next section). For the clinically applied controlled studies included in this review, the virtual fixation planning methods are extracted and summarized (see Section 6).

Some authors also suggest a semi-automated implant or trajectory design, even though this is currently only done in experimental studies. Maratt et al. (2008) proposed a framework to automatically virtually place fixation screws for distal humerus fracture fixation based on the maximization of a constrained objective function. The objective maximizes the number of bicortical screws placed, favoring screws of greater length that cross multiple fracture planes, while avoiding screw collision. In three test cases, the optimal solution was generated in less than a minute. However, no biomechanical analysis was incorporated into the model. Han et al. (2019) developed an SSM based automatic planning tool for pelvic fracture surgery. They developed a pelvic shape atlas using 40 CT scans including an expert definition on safe trajectories within the bone for fixating ten common fracture patterns. Patient-specific planning was obtained by mapping the SSM to the unsegmented patient CT. However, this method does not seem suitable for highly displaced fractures since the SSM would not be able to fit to the displaced shape.

#### 4.3.1 3D printed patient-specific implants and pre-contouring of plates

The patient-specific implants that are used for virtual fixation are usually not readily available. In these cases, it is possible to either 3D print the designed implants or to pre-contour existing implants on a 3D printed reduced bone model. One study by Tomaževič et al. (2021) reported on the use of 3D printed implants made of polyamide, but this was only applied on plastic bone models with acetabular fractures and not in patients. Metal 3D printing of implants is also possible and was done by Wang et al. (2020a). However, pre-contouring of existing titanium osteosynthesis plates is much more common and has been frequently applied clinically (Li et al., 2015, 2019; Zeng et al., 2016; Castro-Núñez et al., 2018; Maini et al., 2018; Chen et al., 2019; Hung et al., 2019; Marschall et al., 2019; Huang et al., 2020; Onodera et al., 2020; Agarwal et al., 2021).

Using conventional methods, osteosynthesis plates used for the fixation of fractures often require multiple intraoperative contouring maneuvers to fit the individual adequately (IJpma et al., 2021). This repeated bending of plates decreases the strength of the plates and might lead to re-displacement of fractures because of an unsatisfactory fit (Maini et al., 2018; Chen et al., 2019). Additionally, it may take a long time to shape the plates intra-operatively. Pre-bending or pre-contouring of implants prior to surgery or 3D printing of patient-specific implants mitigate these issues, and might lead to more accurate reduction, shorter operation times, less tissue stripping and devascularization of fragments and less bleeding (Li et al., 2015; Nasr et al., 2021). The clinical effects of pre-contouring will be further evaluated in Section 7. One consideration regarding the 3D printing or pre-contouring of implants is the fact that the simulation, printing, (pre-contouring) and sterilization may take up to 36 h (Teo et al., 2021). In some fracture situations, such as acetabular trauma cases, this is no problem, since the surgery is recommended to take place within 5–10 days after the accident (Chen et al., 2019). However, in other cases, delayed operation may lead to fibrous union in the fracture gap, which further complicates the surgery (Li et al., 2015).

### 4.4 Analysis virtual bone fracture reduction and fixation

In some studies, the quality of the planned reduction and fixation is analyzed. Typically, only the geometrical quality is analyzed. This might include an evaluation of the achieved reduction, the fit of the implant and the screw trajectories, either qualitatively or quantitatively. Only very few systems evaluate the biomechanical quality of the chosen fixation approach. Since the bone healing response and outcome is highly dependent on the biomechanical environment within the fracture (Perren and Rahn, 1980), this is highly relevant. The fixation should provide a condition with inter-fragmentary strains between 2% and 10%, which is optimal for callus formation (Perren, 1989; Chung, 2018). The biomechanical analysis can be conducted using FE Analysis (FEA), which allows for the numerical computation of displacements, strains and stresses throughout the bone and implant (Poelert et al., 2013). FEA is currently not included in any of the commercially available VSP systems but is occasionally performed using separate numerical software, such as ABAQUS (Dassault Systems, USA) or Ansys (Canonsburg, Pennsylvania, USA) both experimentally (Harith et al., 2016; Shim et al., 2017; Chung, 2018; Mortazavi et al., 2019; Aubert et al., 2021) as well as clinically (Moldovan et al., 2020; He et al., 2022). He et al. (2022) combined FEA with computer-assisted preoperative planning for distal femoral fractures and found that comparing the stress and deformation of different plate-screw combinations provided relevant support for clinicians to select the optimal biomechanical conditions especially in the selection of appropriate plate length and screw positioning. However, building a patient-specific FE model requires the patient-specific bone geometry, assignment of material properties, implant configuration and boundary conditions, implant-bone interaction definitions, and application of a postoperative loading profile, which is rather time-consuming and requires expert knowledge. Additionally, the simulations themselves are computationally expensive. The amount of new information obtained in less complicated fractures might not be worth this additional time and effort (Moldovan et al., 2020). Aubert et al. (2021) developed a patient-specific FEA approach for the surgical planning of tibial plateau fractures. They simulated four fixation scenarios in three different healing conditions (mobile, bonded and fused) for one patient, resulting in twelve different scenarios, and evaluated the mechanical strength, stress distributions in the bone and implants and inter-fragmentary strains and fragment kinematics. They developed the workflow postoperatively but mention that it could provide worthwhile information for preoperative planning tasks to choose the optimal stabilization methods. Harith et al. (2016) developed a framework to automatically simulate the placement and postoperative deformation of an optimally fitting osteosynthesis plate. However, the proposed algorithm requires an intact bone rather than a bone with fracture fragments. Chung (2018) developed and validated a framework to preoperatively simulate alternative plate designs and materials for bone fracture fixation. However, they used a simplified analytical model based on a modifiable parametric model, which was not based on an individual patient. They do state that the framework could be adapted to patient-specific CT-based models.

Theoretically, it is also possible to simulate the patient-specific healing response following a certain fixation scheme. By coupling the mechanical signals outputted by the FEA to changes in biological parameters using partial differential equations or agent-based models, the bone regeneration process can be simulated (Borgiani et al., 2017). However, to the authors’ knowledge, this has never been implemented in a VSP framework.

### 4.5 Intra-operative navigation

After VSP, it is possible to use aiding techniques to guide the surgery according to the planning. This is termed intra-operative navigation and can be conducted by means of image fusion with intra-operative fluoroscopy. However, this is outside the scope of this review and has already been extensively reviewed by other authors including Vetter et al. (2014) and Székely et al. (2016).

Patient-specific instrumentation (PSI) such as CAD drilling guides, CAD implants or pre-contoured implants may also be used to make sure the surgery is performed according to the preoperative planning. Using patient-specific or pre-contoured implants, it can be ensured that the planned reduction is achieved, since the implants are designed in such a way that they optimally fit the reduced bone. An alternative is to use patient-specific drilling guides to translate the planned screw trajectories towards the actual surgery. It has been shown that this permits more accurate and efficient reductions compared with the freehand technique (Schweizer et al., 2016; Merema et al., 2017; IJpma et al., 2021; Long et al., 2021; Nasr et al., 2021).

## 5 Existing software solutions

The 79 studies included in this review were screened for the used software solution. An overview of these systems is given in Table 1. The most commonly used VSP system is the commercial software Materialise Mimics (Materialise, Leuven, Belgium), which was used in 32 clinical studies. This system allows for the three main VSP steps, i.e., construction of patient-specific geometrical models, virtual bone fracture reduction and virtual bone fracture fixation. The used implant for virtual fixation is often designed using Materialise 3-matic (Materialise, Leuven, Belgium), Geomagic (3D systems, Rock Hill, SC, USA) and/or Solidworks (Dassault Systèmes, Waltham, MA, United States) and imported back into Mimics for virtual placement. Another popular VSP system by Materialise is ProPlan CMF, which is developed especially for craniomaxillofacial surgical planning. The planning made using this system is often afterwards imported into a surgical navigation system to guide the surgery. Another common software framework used for the preoperative planning of craniomaxillofacial fractures is a combination of iPlan CMF (BrainLAB, Feldkirchen, Germany) and SurgiCase CMF (Materialise, Leuven, Belgium) for planning and VectorVision (BrainLAB, Feldkirchen, Germany) for intra-operative navigation. Based on the report of the included studies (Westendorff et al., 2006; He et al., 2013; Gong et al., 2017; Dai et al., 2018) using this combination of systems, it is not exactly clear which planning steps can be performed. SuperImage Orthopedics (Cybermed Ltd., Shanghai, China) is a commercial VSP system developed by Chen and Shao (2009), which has been frequently clinically applied for different types of fractures. The system enables the three main VSP steps and features an implant database to use for virtual fixation planning. The system allows for semi-automatic segmentation and reduction by means of manual selection of three points on each fragment and subsequently, the required transformation is automatically computed. However, this VSP system is only available in Chinese, which complicates widespread adoption. Another clinically applied software solution worth highlighting is the patient-specific biomechanical modelling (PSBM) framework developed by Boudissa et al. (2014) for hip fracture fixation planning. The framework uses the existing non-commercial software ITK-snap (Philadelphia, PA, USA), ArtiSynth (ArtiSynth, Vancouver, Canada) and CamiTK (Grenoble, France) to perform semi-automatic geometrical model construction and virtual fragment reduction. The virtual reduction is performed using a biomechanical simulation model where bone fragments are constrained by ligaments, muscles and user interactions including surgical instruments. The rationale of this approach is that the surgical reality is mimicked so that the procedure can be practiced. To the authors’ knowledge, this is the only clinically applied biomechanical simulator for fracture surgery planning. Another in-house software solution is Zed-Trauma (LEXI Co., Ltd., Tokyo, Japan), which was developed by Tokoti and Yoshii et al. (2017) and has been frequently applied by the same authors for radial and humeral fracture fixation planning. The software allows for all three typical VSP steps and features an implant database consisting of plates and screws that can be adjusted in size for virtual placement. After virtual reduction and fixation, the shape can be analyzed using measuring tools incorporated in the software. However, this software is only available in Japanese. A last software solution worth mentioning is CASPA (CARD AG, Zurich, Switzerland) which allows for the three typical VSP steps but has some additional features. The software allows for incorporation of the healthy contralateral bone to guide virtual reduction and enables automatic ICP registration of the fragments to this template. Additionally, patient-specific implants can be designed in this software and the software incorporates geometrical measuring tools for preoperative analysis of the planned reduction.

**TABLE 1:** VSP systems used in clinical practice.

System	Used in references	Used for fracture types
Materialise Mimics (Materialise, Leuven, Belgium	Hu et al., 2011; Liu et al., 2013, Liu et al., 2017; Yang et al., 2013; El-Gengehi and Seif, 2015; Huang et al., 2015, Huang et al., 2020; Li et al., 2015, Li et al., 2019; Zhang et al., 2015, Zhang et al., 2021; Wang et al., 2016a, Wang et al., 2020b, Wang et al., 2020a; Zeng et al., 2016; Merema et al., 2017; Castro-Núñez et al., 2018; Dreizin et al., 2018; Maini et al., 2018; Sinha et al., 2018; Chen et al., 2019; Hung et al., 2019; Marschall et al., 2019; Mishra et al., 2019; Liang et al., 2020; Ramanathan et al., 2020; Agarwal et al., 2021; IJpma et al., 2021; Nasr et al., 2021; Duran-Rodriguez et al., 2022; He et al., 2022; Zheng et al., 2022	Total (*n* = 32): Pelvic/Acetabular fractures (*n* = 14), Craniomaxillofacial fractures (*n* = 12), Tibia fractures (*n* = 3), Humerus fractures (*n* = 1), Calcaneal fractures (*n* = 1), Other (*n* = 1)
+ Materialise 3-matic (Materialise, Leuven, Belgium)	Chen et al., 2019; Marschall et al., 2019; Mishra et al., 2019; Agarwal et al., 2021; IJpma et al., 2021; Zhang et al., 2021; Duran-Rodriguez et al., 2022	
+ Geomagic (3D systems, Rock Hill, SC, United States	Huang et al., 2015; Merema et al., 2017; Wang et al., 2020b; Liang et al., 2020; Ramanathan et al., 2020; IJpma et al., 2021; He et al., 2022	
+ SolidWorks (Dassault Systèmes, Waltham, MA, United States)	Zhang et al., 2015; Merema et al., 2017; Wang et al., 2020a; IJpma et al., 2021; He et al., 2022	
ProPlan CMF (Materialise, Leuven, Belgium)	Cornelius et al., 2015; Wilde et al., 2015; Kokosis et al., 2018; Maloney and Rutner, 2019; Yao et al., 2019; Jie et al., 2020; Cheng et al., 2021	Craniomaxillofacial fractures (*n* = 7)
iPlan CMF (BrainLAB, Feldkirchen, Germany) + SurgiCase CMF (Materialise, Leuven, Belgium) + VectorVision (BrainLAB, Feldkirchen, Germany)	Westendorff et al., 2006; He et al., 2013; Gong et al., 2017; Dai et al., 2018	Craniomaxillofacial fractures (*n* = 4)
SuperImage Orthopedics Edition 1.1 (Cybermed Ltd., Shanghai, China)	Chen et al., 2014, Chen et al., 2015, Chen et al., 2018, Chen et al., 2020; Xia et al., 2015, Xia et al., 2019; Wang et al., 2020c; Jia et al., 2020	Total (*n* = 8): Humerus fractures (*n* = 4), Femoral fractures (*n* =3), Calcaneal fractures (*n* = 1)
Zed-Trauma distal radius stage (LEXI Co., Ltd. Tokyo, Japan)	Yoshii et al., 2017, Yoshii et al., 2019b, Yoshii et al., 2019a, Yoshii et al., 2020, Yoshii et al., 2021; Totoki et al., 2018	Total (*n* = 6): Radius fractures (*n* = 5), Humerus fractures (*n* = 1)
D2P (3D Systems, Rock Hill, SC, United States)	Khatib et al. (2018)	Craniomaxillofacial fractures (*n* = 1)
CASPA (CARD AG, Zurich, Switzerland)	Schweizer et al., 2016; Casari et al., 2021; Zindel et al., 2021	Total (*n* = 3): Radius fractures (*n* = 2), Scaphoid fractures (*n* = 1)
TraumaTech (3DIM, Ostrava, Czech Republic)	Madeja et al. (2021)	Scapula fractures (*n* = 1)
E3D Digital (Hunan province, China)	Long et al. (2021)	Femur fractures (*n* = 1)
Democratiz3D (Embodi3d, Washington, United States)	Moldovan et al. (2020)	Tibia fractures (*n* = 1)
PSBM (Boudissa *et al,* France)	Boudissa et al., 2018; Boudissa et al., 2021a; Boudissa et al., 2021b	Acetabular/Pelvic fractures (*n* = 3)
SQ Pelvis (Cimerman and Kristan*,* Slovenia)	Cimerman and Kristan, (2007)	Acetabular/Pelvic fractures (*n* = 1)
Osteo3D (Bengaluru, India)	Chakravarthy et al. (2019)	Craniomaxillofacial fractures (*n* = 1)
FreeForm (Sensable, Wilmington, United States)	Onodera et al. (2020)	Craniomaxillofacial fractures (*n* = 1)
Simpleware ScanIP (Synopsys, NC, United States)	Ma et al. (2017)	Craniomaxillofacial fractures (*n* = 1)
M-3D software (Forward Algorithm Company, Shanghai, China)	Wang et al. (2016b)	Acetabular fractures (*n* = 1)
TraumaCAD (Orthocrut, Tel Aviv, Israel)	Pilson et al. (2008)	Tibia fractures (*n* = 1)
IPS CaseDesigner Software (KLS Martin group, Jacksonville, Florida, United States).	Badiali et al., 2020; Kongsong and Sittitavornwong, 2020	Craniomaxillofacial fractures (*n* = 2)
Blender + OrtogOnBlender (Amsterdam, the Netherlands)	de Carvalho et al. (2021)	Craniomaxillofacial fractures (*n* = 1)
Other (self-designed / combination of open-source software)	Fornaro et al., 2010a; Vetter et al., 2018; Oki et al., 2021	Total (*n* = 3): Radius fractures (*n* = 1), Acetabular/pelvic fractures (*n* = 1), Fibula fractures (*n* = 1)

## 6 Clinical feasibility

Of the 21 included studies that compare conventional planning with computer-assisted planning, the study characteristics, investigated VSP systems, fracture classification and used VSP approaches are summarized in Table 2. The required input data, segmentation methods and duration, virtual reduction methods and duration, virtual fixation duration and analysis duration, of these 21 studies are summarized in Tables 3, 4. All of the included studies require a (thin-slice) CT scan of the fractured bone and subsequently perform semi-automatized segmentation methods. The most common reduction method is by manual mouse interaction, but some studies use a semi-automated approach. The mean total time required for the planning, including segmentation and virtual reduction, ranges from 22 to 258 min in different studies. When 3D printing and pre-contouring are performed, the entire process may take up to 1,038 min.

**TABLE 2:** Study characteristics of included studies with a control group. (1) 3D reconstruction (2) virtual reduction, (3a) virtual fixation with implant from library, (3b) virtual fixation with CAD implant (4a) Geometrical analysis, (4b) FEA, (5a) 3D printing of CAD implant, (5b) CAD and 3D printing of guide plates (5c) 3D printing reduced/mirrored bone model + pre-contouring.

Authors	Country	Study design	Study Period	N (VSP /control	Fracture type	Planning methods	Investigated VSP system(s)
He et al. (2022)	China	RCT	2017–2020	*N* = 31 (16/15)	Femur (distal)	(1-3a), (4b)	Materialise Mimics Medical 21.0 for (1-2) + SolidWorks for (3) + Ansys 19.0 for (4b)
Zheng et al. (2022)	China	Retrospective case-control study	2014–2018	*N* = 45 (24/21)	Acetabulum (posterior wall, comminuted)	(1-3a)	Materialise Mimics 20.0
Long et al. (2021)	China	Prospective cohort study	2019–2020	*N* = 40 (20/20)	Femur (neck)	(1-3b), (5b)	E3D Digital Medical modeling and design
Boudissa et al. (2021b)	France	Prospective cohort study	2019	*n* = 22 (10/12)	Acetabulum	(1-2) using biomechanical simulation model (bone fragments constrained by ligaments, muscles, and user interactions incl. surgical instruments)	In-house developed system. ITK-Snap + MeshLab for (1), Artisynth Java to manage mechanics, user interaction and visualization in CamiTK C++ (2)
Boudissa et al. (2021a)	France	Retrospective case-control study	2015–2019	*n* = 30 (10/20)	Acetabulum	(1-2) using biomechanical simulation model (bone fragments constrained by ligaments, muscles, and user interactions incl. surgical instruments)	In-house developed system. ITK-Snap + MeshLab for (1), Artisynth Java to manage mechanics, user interaction and visualization in CamiTK C++ (2)
Wang et al. (2020b)	China	Retrospective cohort study	2012–2015	*n* = 125 (53/72)	Femur (intertrochanteric, geriatric)	(1-3a), (4a)	SuperImage Orthopedics Edition 1.0
Wang et al. (2020a)	China	Retrospective cohort study	2016–2017	*n* = 50 (15/35)	Acetabulum (with quadrilateral plate disruption)	(1) + mirroring of uninjured side, (3b), (5a). NB: conventional treatment requires intraoperative plate bending	Materialise Mimics 15.0 for (1) + Geomagic + SolidWorks for (3b)
Jia et al. (2020)	China	Retrospective cohort study	2009–2018	*n* = 1221 (465/756)	Femur (intertrochanteric)	(1-3a)	SuperImage Orthopedics Edition 1.1
Huang et al. (2020)	China	RCT	2013–2017	*n* = 40 (20/20)	Acetabulum (both-column)	(1-2), (5c). NB: conventional treatment requires intraoperative plate bending	Materialise Mimics 15.0 for (1-2) + Materialise Magics 21.0 for 3DP (5c) support
Ramanathan et al. (2020)	India	RCT	2017–2019	*n* = 30 (15/15)	Mandible (displaced, maloccluded)	(1-3b) (5a). NB: conventional treatment requires fabrication of splint using dental stone of patient’s dentition.	Materialise Mimics for (1) + Geomagic for (2-3b)
Chen et al. (2020)	China	Retrospective cohort study	2014–2017	*n* = 32 (15/17)	Femur (distal)	(1-3a)	SuperImage Orthopedics Edition 1.0
Yoshii et al. (2019)	Japan	Prospective cohort study	2014–2019	*n* = 60 (30/30)	Radius (distal)	(1-3a). NB: conventional planning based on radiographs only	Zed-Trauma distal radius stage
Li et al. (2019)	Taiwan	Retrospective cohort study	2013–2017	*n* = 16 (7/9)	Acetabulum (posterior wall/column, with hip dislocation)	(1) + mirroring of uninjured side, (5c)	Materialise Mimics
Hung et al. (2019)	Taiwan	Retrospective cohort study	2012–2017	*n* = 30 (16/14)	Pelvis (anterior ring)	(1-2), (5c) NB: conventional treatment requires intraoperative plate bending	Materialise Mimics 19.0
Chen et al. (2019)	China	Retrospective cohort study	2013–2017	*n* = 52 (28/24)	Acetabulum (both-column)	(1-3b), (5c). NB: conventional treatment requires intraoperative plate bending	Materialise Mimics 16.0 for (1-2) + Materialise 3-matic for (3b), Cura Ultimaker for 3DP (5c) preparation
Totoki et al. (2018)	Japan	Prospective cohort study	Not specified	*n* = 49 (30/19)	Radius (distal)	(1-3a)	Zed-Trauma distal radius stage
Maini et al. (2018)	India	RCT	2014-2016	*n* = 25, (12/13)	Acetabulum (displaced)	(1-3b), (5a: PLA) + pre-contouring titanium plates on PLA plates. NB: conventional treatment requires intraoperative plate bending	Materialise Mimics for (1-2) + Materialise 3-matic for (3b-5a)
Chen et al. (2018)	China	Retrospective cohort study	2009–2015	*n* = 131, (46/53/32*). *3D printing group	Humerus (proximal, displaced, three-/four-part)	(1-3a)	SuperImage Orthopedics Edition 1.0
Liu et al. (2017)	China	RCT	2010–2012	*n* = 32 (16/16)	Tibia (diaphyseal)	Planning method not clearly specified, based on figures in study: (1) 3D reconstruction contralateral tibia, (3) virtual fixation	Materialise Mimics
Zhang et al. (2015)	China	RCT	2011–2013	*n* = 32 (14/18)	Tibia (plateau, Schatzker type III)	(1-3b)	Materialise Mimics 10.01 for (1-2), SolidWorks for (3b)
Li et al. (2015)	China	RCT	2010–2013	*n* = 24, (12/12)	Mandible (comminuted)	(1-2), (5c). NB: conventional treatment requires intraoperative plate bending	Materialise Mimics 10.01

**TABLE 3:** Simulation methods used in 21 included studies. -: Not specified. N/A: Not applicable.

Authors	Input data	Segmentation method	Virtual reduction method
He et al., 2022	CT (- mm)	Thresholding, region growing, manual adjustments	—
Zheng et al., 2022	CT (1 mm)	Thresholding, manual adjustments	Manual (mouse)
Long et al., 2021	CT (- mm)	—	—
Boudissa et al., 2021b	CT (‘high-resolution')	Thresholding, region growing, manual adjustments	Manual (mouse)
Boudissa et al., 2021a	CT (‘high-resolution')	Thresholding, region growing, manual adjustments	Manual (mouse)
Wang et al., 2020c	CT (‘thin-slice')	—	Semi-automatic: manual selection 3 points each fragment
Wang et al., 2020a	CT (1 mm)	Thresholding (min226 - max1476)	N/A
Jia et al., 2020	CT (- mm)	—	—
Huang et al., 2020	CT (- mm)	Thresholding + manual adjustments	Manual (mouse)
Ramanathan et al., 2020	CT (0.6 mm) + intraoral scanning	—	—
Chen et al., 2020	CT (‘thin-slice')	Semi-automatic	Semi-automatic: manual selection 3 points each fragment
Yoshii et al., 2019b	CT (1 mm)	“Cut function"	—
Li et al., 2019	CT (3 mm)	Variant thresholding	N/A
Hung et al., 2019	CT (3 mm)	—	—
Chen et al., 2019	CT (1 mm)	Manual	Manual (mouse)
Totoki et al., 2018	CT (1 mm)	“Cut function"	—
Maini et al., 2018	CT (1 mm)	Thresholding, region growing, manual adjustments	Manual (mouse)
Chen et al., 2018	CT (- mm)	Semi-automatic	Semi-automatic: manual selection 3 points each fragment
Liu et al., 2017	CT (1–1.5 mm) - bilateral	—	—
Zhang et al., 2015	CT (1 mm)	Thresholding, manual adjustments	—
Li et al., 2015	CT (0.5 mm)	Thresholding, manual adjustments	Manual (mouse)

**TABLE 4:** Simulation duration reported in included studies. Studies are only included in the table if times were reported. Time is reported as mean ± standard deviation or as a median (25—75%). -: Not specified. N/A: Not applicable.

Authors	Segmentation time (min)	Reduction time (min)	Fixation time (min)	Analysis time (min)	Total VSP time (min)	3D printing time (min)	Pre-contouring time (min)
He et al., 2022	38.12 ± 8.83	18.89 ± 6.89	73.23 ± 9.94	64.06 ± 5.21	194.29 ± 31.81	N/A	N/A
Boudissa et al., 2021b	82 ± 18	22 ± 4	N/A	N/A	104 ± 18	N/A	N/A
Boudissa et al., 2021a	83 ± 18	23 ± 4	N/A	N/A	105 ± 18	N/A	N/A
Wang et al., 2020c	6.89 ± 2.55	13.49 ± 3.25 for reduction + fixation		4.50 (2.00, 6.00)	24.73 ± 4.01	N/A	N/A
Jia et al., 2020	—	—	—	N/A	27.0 ± (…)	N/A	N/A
Chen et al., 2020	8—11^*^	5—23^*^	10–18^*^	N/A	40.4 ± 11.7^†^	N/A	N/A
Li et al., 2019	—	N/A	N/A	N/A	11.14 ± 1.07	608.43 ± 27.54	46.86 ± 17.69
Hung et al., 2019	—	—	N/A	N/A	46.56 ± 22.78	929.06 ± 206.38	62.50 ± 21.45
Chen et al., 2019	—	—	-	N/A	120 ± (…)	720 ± (…)	N/A
Maini et al., 2018	—	—	—	N/A	258 (120–420)	—	—
Chen et al., 2018	—	—	—	N/A	22–45	N/A	N/A
Zhang et al., 2015	—	—	—	N/A	43.3 ± 8.2	N/A	N/A
Li et al., 2015	—	—	N/A	N/A	30 ± (...)	240–320 for printing + pre-contouring	

* Values read from graph.

† Recalculated from three fracture subgroups.

Wang D. et al. (2020) and Jia et al. (2020) have also assessed the learning curve for clinicians associated with computer-assisted planning compared to conventional planning for intertrochanteric hip fracture fixation surgery. They assessed the learning curve in terms of surgery duration, blood loss, and number of fluoroscopic images performed against number of patients and found that the computer-assisted planning approach led to a less steep learning curve.

## 7 Clinical efficacy: Clinical outcomes

**FIGURE 4:**
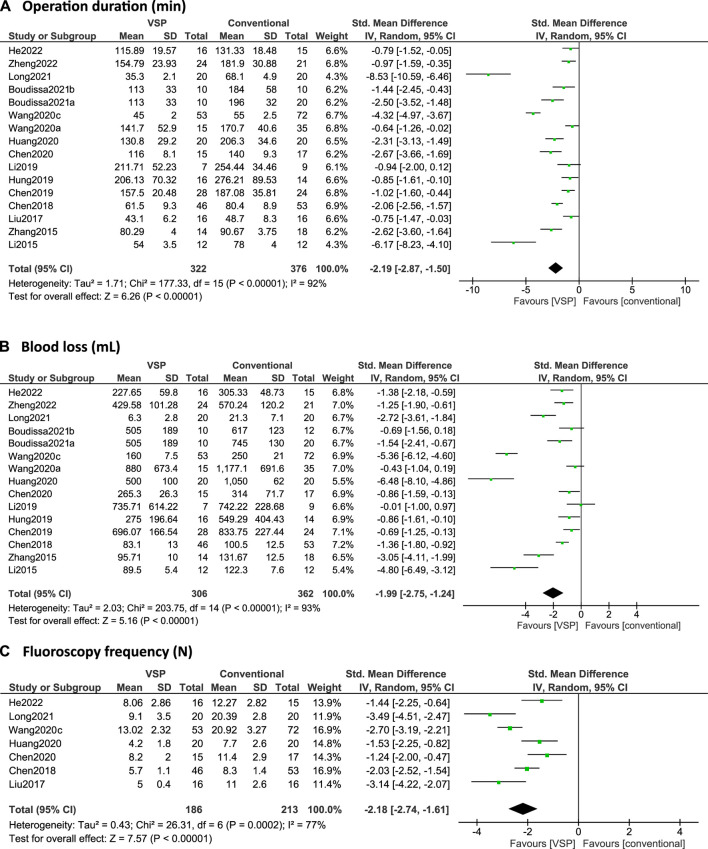
**(A–C)**: Forest plots of intraoperative clinical outcomes. Green boxes + horizontal lines: standardized mean differences as reported by the individual studies along with 95% confidence intervals, size of the box represents the weight of the individual studies. Diamonds: combined standardized mean difference with the outer edges representing the 95% confidence interval. Total: number of patients. **(A)** Operation duration (min), **(B)** Blood loss (ml), **(C)** Intra-operative fluoroscopy frequency (N).

**FIGURE 5:**
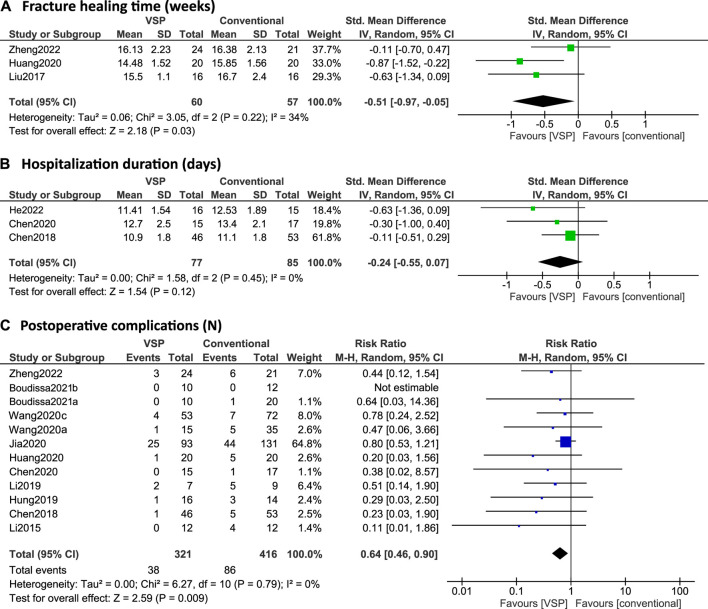
**(A–C)**: Forest plots of postoperative clinical outcomes. Green/blue boxes + horizontal lines: standardized mean differences **(A/C)** or risk ratios **(B)** as reported by the individual studies along with 95% confidence intervals, size of the box represents the weight of the individual studies. Diamonds: combined standardized mean difference **(A/C)** or risk ratio **(B)** with the outer edges representing the 95% confidence interval. Total: number of patients. **(A)** Fracture healing time (weeks), **(B)** Hospitalization duration (days), **(C)** Postoperative complications (N).

In Figures 4A–C and Figures 5A–C, forest plots are shown of the intraoperative outcomes (operation time, blood loss, fluoroscopy frequency) and postoperative outcomes (fracture healing times, hospitalization duration and complication rates) respectively, as reported by the included studies. Studies are only included in the figures if the outcome measures were reported as mean plus standard deviation. Additionally, reduction quality and functional scores are summarized in Table 5.

**TABLE 5:** Reduction quality difference and functional score difference reported in included studies. -: Not reported. N.S: not statistically significant. S.D*/*: statistically significant difference.

Authors	Reduction score (scoring system)	Functional score (scoring system)
He et al., 2022	—	N.S. (VAS score, Knee society score, ROM)
Zheng et al., 2022	N.S. (Matta)	N.S. (Merle d'Aubigne score)
Long et al., 2021	N.S. (Not specified)	N.S. (Harris score)
Boudissa et al., 2021b	N.S. (Matta)	—
Boudissa et al., 2021a	N.S. (Matta)	—
Wang et al., 2020c	—	N.S. (Harris score)
Wang et al., 2020a	N.S. (Matta + residual displacement)	—
Jia et al., 2020	—	N.S. (Harris score, SF-36, PCS score, VAS score)
Huang et al., 2020	80% (VSP) vs 30% (control)* (good reduction rate = displacement <2 mm)	75% (VSP) vs. 30% (control)* (excellent/good rate Harris)
Ramanathan et al., 2020	0 mm (VSP) vs 0.47 mm (control)* (interfragmentary separation)	0.02 (VSP) vs 8.2 (control)* (VAS score)
Chen et al., 2020	—	N.S. (KSS, SF-36, VAS-score, ROM)
Yoshii et al., 2019b	S.D.* (difference between operated and healthy side)	—
Li et al., 2019	N.S. (displacement articular surface)	—
Hung et al., 2019	N.S. (residual displacement)	—
Chen et al., 2019	N.S. (Matta)	N.S. (Merle d’Aubigne score)
Totoki et al., 2018	86.1% (VSP) vs 74.8% (control)* (appropriate screw choices)	—
Maini et al., 2018	3.76 vs 4.09 (Matta, mean residual displacement: mm, no *p*-value)	—
Chen et al., 2018	—	ASES scoring*, Constant-Murley score*, SF-36*, ROM*
Liu et al., 2017	N.S. (lower limb alignment)	N.S. (Johner-Wruhs score)
Zhang et al., 2015	—	—
Li et al., 2015	92% ± 4.2 (VSP) vs 83 % ± 5 (control)* (level of mandibular symmetry)	N.S. (patient satisfaction, interincisal opening)

### 7.1 Operation time

16 studies reported on operation time (Li et al., 2015, 2019; Zhang et al., 2015; Liu et al., 2017; Chen et al., 2018, 2019, 2020; Hung et al., 2019; Wang D. et al., 2020, 2020a; Huang et al., 2020; Boudissa et al., 2021a, 2021b; Long et al., 2021; He et al., 2022; Zheng et al., 2022). All studies, except for Li et al. (2019), found that using computer-assisted planning leads to a significantly shorter operation time compared to conventional planning. Over all the studies, the operation time was significantly shorter for the VSP group, with an overall standardized mean difference (SMD) of -2.19 (95% CI: -2.87, -1.50), see Figure 4A.

### 7.2 Blood loss

15 studies reported on intra-operative blood loss (Li et al., 2015, 2019; Zhang et al., 2015; Chen et al., 2018, 2019, 2020; Hung et al., 2019; Wang D. et al., 2020, 2020a; Huang et al., 2020; Boudissa et al., 2021a, 2021b; Long et al., 2021; He et al., 2022; Zheng et al., 2022) and all studies except for Li et al. (2019) found that using VSP leads to significantly less blood loss. Boudissa et al. (2021a) and Wang et al. (2020b) did report that they found significant differences in blood loss using student t-tests and Mann-Whitney tests respectively, while the 95% CI intervals computed in this review using Review Manager failed to replicate these results, probably because of different test assumptions (see Figure 4B). The meta-analysis shows that overall, VSP leads to significantly less blood loss in comparison with the conventional group with a standardized mean difference of -1.99 (95% CI: -2.75, -1.24), see Figure 4B.

### 7.3 Fluoroscopy frequency

All studies that investigated fluoroscopy frequency (Liu et al., 2017; Chen et al., 2018, 2020; Wang D. et al., 2020; Huang et al., 2020; Long et al., 2021; He et al., 2022) found that using VSP leads to significantly less intra-operative fluoroscopies. The meta-analysis resulted in an overall standardized mean difference of -2.18 (95% CI: -2.75, -1.61), see Figure 4C.

### 7.4 Fracture healing time

Four studies investigated fracture healing time differences between the VSP and conventional planning groups (Liu et al., 2017; Wang D. et al., 2020; Huang et al., 2020; Zheng et al., 2022). However, Wang D. et al. (2020) reported the fracture healing time as median (25%, 75% quartile) with 18.00 (15.50, 19.00) weeks for the VSP group and 18.00 (16.00, 20.00) weeks for the conventional group (*p* > 0.05). Because no clear indications were given that a normal distribution could be assumed, the results were excluded from the meta-analysis, which required means and standard deviations as input. Out of the other three studies, only Huang et al. (2020) found a statistically significant difference between the two groups. Overall, the estimated effect was also significant with a standardized mean difference of -0.51 (95% CI: -0.97, -0.05), see Figure 5A.

### 7.5 Hospitalization duration

Three studies reported on hospitalization duration (Chen et al., 2018, 2020; He et al., 2022). Chen et al. (2020) did report a significant difference in hospitalization days (*p* < 0.05) using a Mann-Whitney test or the Kruskal–Wallis test (not specified) but this result was not reproduced by the meta-analysis in this review, which uses a t-test (see Figure 5B). Overall, no significant difference was found in hospitalization duration with the meta-analysis (SMD: -0.24, 95% CI: -0.55, 0.07), see Figure 5B.

### 7.6 Postoperative complications

12 studies investigated postoperative complications (Li et al., 2015, 2019; Chen et al., 2018, 2020; Hung et al., 2019; Wang M. et al., 2020, 2020a; Huang et al., 2020; Jia et al., 2020; Boudissa et al., 2021a, 2021b; Zheng et al., 2022) and none of them found a significant difference in risk of postoperative complications between the VSP and conventional planning groups (null value RR = 1 within 95% CI for all studies, see Figure 5C). However, when pooling all the reported data for the meta-analysis here, the risk is significantly lower in the VSP group (RR: 0.64; 95% CI: 0.46, 0.90), see Figure 5C.

### 7.7 Reduction quality and functional scores

15 studies reported on reduction quality (Li et al., 2015, 2019; Liu et al., 2017; Maini et al., 2018; Totoki et al., 2018; Yoshii et al., 2019b; Chen et al., 2019; Hung et al., 2019; Wang et al., 2020a; Huang et al., 2020; Ramanathan et al., 2020; Boudissa et al., 2021a, 2021b; Long et al., 2021; Zheng et al., 2022), using different scoring systems, including the Matta scoring system (Matta and Tornetta, 1996), residual displacement (mm) and/or level of alignment compared to the healthy contralateral side. 12 studies reported on functional clinical scores (Li et al., 2015; Liu et al., 2017; Chen et al., 2018, 2019, 2020; Wang D. et al., 2020; Huang et al., 2020; Jia et al., 2020; Ramanathan et al., 2020; Long et al., 2021; He et al., 2022; Zheng et al., 2022) using scoring systems such as the VAS score, Harris score, Merle d’Aubigne score and range of motion (ROM). Since the reported reduction and functional scores are highly heterogeneous between studies or not clearly specified, no meta-analysis was performed on these outcome measures. Instead, Table 5 summarizes whether a significant difference in reduction quality and/or functional score was found by the included studies. Five studies found a significant difference in reduction quality favoring VSP (Li et al., 2015; Totoki et al., 2018; Yoshii et al., 2019b; Huang et al., 2020; Ramanathan et al., 2020) and three studies found a significant difference in functional outcomes favoring VSP (Chen et al., 2018; Huang et al., 2020; Ramanathan et al., 2020).

## 8 Discussion

In this review, five main steps in VSP were identified which include 1) construction of patient-specific geometrical models, 2) virtual bone fracture reduction, 3) virtual bone fracture fixation, 4) analysis of the fixation reduction and/or fixation, and 5) intra-operative navigation. The first three stages are already routinely applied in clinical practice, the fourth stage rarely, and the fifth stage is outside the scope of this review.

Construction of patient-specific geometrical models (stage 1) currently relies on 3D imaging technologies, more specifically on thin-slice CT scans. The segmentation of the bone fragments from the CT scans is usually done using semi-automatic approaches, often relying on intensity thresholding, region growing techniques and manual adjustments. The mean duration of the segmentation ranged from 6.89 to 83 min in the different studies. Many studies are currently focused on automatizing the segmentation of bone, and with the further development of machine learning for these purposes, efficient segmentation methods will hopefully continue to improve (Liu et al., 2021; Verhelst et al., 2021; Deng et al., 2022). However, especially the fracture fragment separation is labor-intensive and harder to automatize. An additional intrinsic drawback of using CT scans (and subsequent segmentation) is the additional radiation exposure for the patient and the fact that making a thin-slice CT scan of the whole bone is not usually part of the clinical routine of bone fracture management. Future efforts should therefore focus on either reducing the radiation exposure associated with CT scans (e.g., using EOS 2-D/3-D image systems (Takao et al., 2018b)) or employing alternative methods of patient-specific geometrical model construction (e.g., using SSMs).

Virtual bone fracture reduction (stage 2) currently usually relies on manual rotation and translation of the bone fragments using mouse interaction. Some studies employed a semi-automatic approach, requiring manual selection of points on each fragment and subsequent automatized reduction of the fragments. The mean time required for virtual reduction ranged from 5 to 23 min in the different studies. However, many experimental systems are currently investigating more automatized reduction methods with promising results.

Implants used for virtual bone fracture fixation (stage 3) are usually either chosen from an implant database and scaled or designed for the specific patient using CAD tools. The mean reported times required for (design and) implantation of the fixation device range from 10 to 73.23 min in the different studies.

Analysis of the planned surgery (stage 4) is currently rarely done and when done, it mainly relies on a geometrical evaluation (Wang D. et al., 2020), ignoring biomechanical aspects. The biomechanical conditions are known to influence healing outcome and therefore could be used to support clinical decision-making. Several studies have investigated the mechanical conditions within bone fractures using FE techniques and their relation to healing outcome (Shefelbine et al., 2005; Kim et al., 2011; Taylor and Prendergast, 2015; Matsuura et al., 2017; Dailey et al., 2019; Lewis et al., 2021; Schwarzenberg et al., 2021). This knowledge could be incorporated into VSP systems and used for the development of improved personalized implants and better surgeon education, possibly resulting in fewer non-union and fixation failures. However, FE models of fractured bones are computationally expensive and require labor-intensive work. The mean time required for the FE analysis reported by He et al. (2022) was 64.06 ± 5.21 min. Therefore, there is a need to develop quicker patient-specific FE models to incorporate biomechanical analysis into the planning routine.

Another aspect to consider regarding the existing VSP approaches is the fact that soft tissues are usually not considered (except by Boudissa et al. (2021a); Boudissa et al. (2021b)). Since the soft tissues interfere with the possible fragment manipulations and impact the biomechanics of the entire system, this is relevant to consider in future frameworks. Omission of the soft tissues from the models might also lead to differences between the preoperative planning and intraoperative implementation (He et al., 2022).

Even though the mentioned aspects of the current VSP frameworks require improvements in the future, this review reveals that the current frameworks are feasible to be used in clinical practice and already significantly improve clinical outcomes. It should, however, be noted that this current review is limited by the nature of the included studies, which should be considered while interpreting these results. First, given the retrospective design of most of the included studies, there is a risk for selection bias and confounding bias. Additionally, division into the VSP and control groups was often not based on randomization but rather on the preferences of the patient. Secondly, many different fracture types are considered in this study, which makes it hard to compare and pool the results. The complexity of the fractures has a considerable influence on the considered clinical outcomes such as intra-operative bleeding and operation time. Additionally, the time required for planning differs greatly between fracture types. For this reason, the standardized mean difference (Cohen’s d) was chosen as an outcome measure in the meta-analysis, but this outcome measure is harder to interpret. Thirdly, potential bias due to differences in skill levels of the surgeons could exist and it is unclear whether the demonstrated benefits will also easily translate to other surgeons. As an example, Chen et al*.* have performed multiple studies on the effects of VSP on surgical outcomes and the planning and actual surgery were always performed by the senior author who had the opportunity to improve over the course of 7 years. However, it is likely that the technology is relatively easy to adopt by different surgeons with different technical backgrounds as demonstrated by Zindel et al. (2021), Jia et al. (2020) and Wang D. et al. (2020). Lastly, a question that remains is whether VSP will prove to be cost-effective and whether the demonstrated clinical benefits are clinically relevant.

An alternative to virtual surgical planning is the use of 3D printed hand-held fracture models. Chen et al. (2018) compared the three planning methods with each other: conventional planning, VSP and 3D printing assisted planning. They found that the clinical outcomes in both the virtual surgical and 3D printing groups were better than those in the conventional group regarding operative time, blood loss, fluoroscopic images, and functional outcome. However, virtual surgical technology had some advantages over 3D printing technology regarding the shorter time for preoperative planning, the interval from injury to surgery and duration of hospital stay.

## 9 Conclusion

In conclusion, computer-assisted preoperative planning of bone fracture fixation surgery has been shown to be feasible to be employed in the clinical routine and to improve intra-operative efficiency in terms of operation time, blood loss, and fluoroscopy frequency. Additionally, using VSP yields shortened fracture healing times and less postoperative complications compared to conventional planning. Some studies have also reported improved reduction quality and functional outcomes. Moreover, VSP has the potential to provide biomechanical feedback on fracture stability, known to highly influence the healing outcome. Future efforts should focus on developing more efficient frameworks that also incorporate biomechanical analyses.

## Footnotes

1 Some identified studies that were not clinically applied were used for Section 4 in a non-systematic manner.

2
https://www.slicer.org/.

3
https://imagej.nih.gov/ij/.
